# Are parental changes related to improvements in preschool children's disruptive behaviours?

**DOI:** 10.1002/cpp.2402

**Published:** 2019-12-04

**Authors:** Lianne van der Veen‐Mulders, Pieter J. Hoekstra, Maaike H. Nauta, Barbara J. van den Hoofdakker

**Affiliations:** ^1^ Department of Child and Adolescent Psychiatry, University Medical Center Groningen University of Groningen Groningen the Netherlands; ^2^ Department of Clinical Psychology and Experimental Psychopathology University of Groningen Groningen the Netherlands

**Keywords:** preschoolers, disruptive behaviour, parenting, behavioural parent training

## Abstract

**Objective:**

To investigate whether changes in parenting after behavioural parent training in routine clinical care are associated with improvements in preschool children's disruptive behaviours.

**Method:**

We evaluated changes after parent training in maternal and paternal self‐reports of parental discipline practices parenting sense of competence, and parents' ratings of child disruptive behaviours in parents of 63 children, with a one group pretest–posttest design. We also compared parenting parameters in this clinical sample with a nonclinical sample (*n* = 121).

**Results:**

Mothers' self‐reports of parental discipline practices and parenting sense of competence significantly improved after behavioural parent training. Less over‐reactivity in both mothers and fathers was associated with fewer disruptive behaviours in children. After parent training, mothers' ratings of their discipline techniques did not differ anymore from those in the nonclinical sample.

**Conclusion:**

Positive changes in parental discipline practices, particularly less over‐reactive parental behaviours, were related to a decrease of disruptive child behaviours.

**Key Practitioner Message**

- Mothers' parental discipline practices and parenting sense of competence improve after behavioural parent training in routine care.
- Fathers' parental discipline practices do not improve after behavioural parent training and fathers' parenting sense of competence only slightly improves
- A reduction in authoritarian, emotional, and harsh parenting after behavioural parent training is associated with improvement of the child's disruptive behaviour problems
- Adaptations of existing parent training programs to the specific needs of fathers may be required

## 1 INTRODUCTION

Behavioural parent training aims to enhance prosocial behaviours and reduce disruptive behaviours in children, by improving parenting practices and decreasing coercive parent–child interactions. A number of studies have indicated that parent training is an effective intervention for preschool children with problem behaviour (Charach et al., 2013; Daley et al., 2017; Kaminski & Claussen, 2017; Mulqueen, Bartley, & Bloch, 2013; Rimestad, Lambek, Zacher Christiansen, & Hougaard, 2016) and that parenting skills and parenting sense of competence improve after behavioural parenting training (Charach et al., 2013; Rimestad et al., 2016). However, we do not yet know if improvements in child behaviour are directly associated with changes in parenting.

A better understanding of the extent to which changes in parenting and improvement in child behaviour are related may be a first step towards developing individually tailored parent training. If indeed improvements in parent training are associated with a reduction of the child's disruptive behaviour, then measuring the extent to which parenting practice improves during the training may be a basis for individualizing the parent training. A clinician may choose to use different interventions, informed by the degree of improvement in parenting skills and child behaviours, rather than applying a standard number of sessions.

The influence of changes in parenting style on children's behaviour may be different for fathers and mothers, because they may have different roles. Generally, mothers are the primary caretakers who spend most of the time with the children during daily routines, while fathers spend most of the time with the children during free time and sports. It may be that fathers recognize behaviour problems in their children, but see mothers as primarily responsible for the child's behaviour (Fletcher, Freeman, & Matthey, 2011). It is therefore relevant to investigate the relation of changes in parenting behaviour in both mothers and fathers with child outcomes after parent training.

In our previous study (Van der Veen‐Mulders, Hoekstra, Nauta, & van den Hoofdakker, 2018), we reported on the effectiveness of behavioural parent training for preschool children with disruptive behaviours, and on parental predictors of response. Both mother and father reported child behaviour problems decreased significantly after treatment. In this secondary analysis, we report on changes in parental discipline practices and sense of competence of both mothers and fathers after behavioural parent training, and possible associations with improvements in young children's disruptive behaviours. Given that inadequate parental disciplining is related to children's disruptive behaviours (Harvey, Metcalfe, Herbert, & Fanton, 2011; Rinaldi & Howe, 2012) and low levels of maternal self‐efficacy is associated with maternal coercion (Bor & Sanders, 2004), we hypothesized that improvements of parental discipline practices and parental sense of competence on parenting through parent training may lead to fewer disruptive child behaviours.

There is some preliminary support that improvements in parental discipline techniques are related to improvements of child conduct problems after parent training, with more support in younger than in older children and more support in prevention studies compared to intervention studies (Forehand, Lafko, Parent, & Burt, 2014). Positive parenting skills, such as environmental restructuring, modelling, planning ahead, and praising are considered to be preventive for the development of disruptive behaviour problems (Cunningham & Boyle, 2002). A study in preschool children with conduct problems found that improvement of positive parenting skills, but not reduction of harsh and negative parenting, mediated the improvement in children's problem behaviour (Hess, Teti, & Hussey‐Gardner, 2004). Furthermore, young children have been shown to respond more favourably to parent training if there were clear improvements in parenting practices (Beauchaine, Webster‐Stratton, & Reid, 2005; Elizur, Somech, & Vinokur, 2017).

Also, improvement of parental self‐efficacy may be associated with better outcome of parent training. Among families of 2‐year‐olds it was found that improvements in mothers' reports of self‐efficacy were significantly associated with improvements in child behaviour after behavioural parent training, but no such associations were found among fathers (Gross, Fogg, Garvey, & Julion, 2004). A study on a prevention program for preschool children with disruptive behaviour problems showed that changes in child behaviour were mediated by changes in negative parenting behaviour, but not by changes in parental self‐efficacy (Hanisch, Hautmann, Plück, Eichelberger, & Döpfner, 2014). However, research on associations between changes in parental discipline strategies and parenting sense of competence with child outcomes after parent training is scarce, especially with respect to referred children. Also, little is known about the influence of changes in paternal parenting behaviours on child outcomes

The main aim of the present study was to examine whether changes in parental discipline practices and parenting sense of competence of mothers and fathers of regularly referred preschool children after behavioural parent training, as provided in routine clinical practice, were associated with changes in parent‐rated child behaviour problems. We hypothesized that improvements in parental discipline strategies and sense of competence would be associated with positive changes in child behaviour after parent training. As a secondary aim, we investigated whether parents reported less adequate parental discipline practices and lower parenting self‐esteem before treatment than parents of preschool children from the general population. Consistent with previous studies (Lorber & Slep, 2015; Wittkowski, Dowling, & Smith, 2016), we expected parents in the nonclinical sample to rate more adequate discipline practices and higher levels of parenting sense of competence. Furthermore, we explored whether fathers and mothers differed on self‐reported parenting parameters before treatment. Concerning differences on parenting between fathers and mothers, one could hypothesize that mothers of young children with disruptive behaviours show lower levels of sense of competence on parenting and less adequate parenting behaviours than fathers, assuming they focus more on daily care and discipline in their parenting role.

## 2 METHOD

### 2.1 Design

To enhance the ecological validity, we performed the study at our outpatient clinic for child‐ and adolescent mental health, in which parents of young referred children with disruptive behaviour problems were offered behavioural parent training as the first treatment step of routine care. In this study we evaluated changes in parental discipline practices, parenting sense of competence and externalizing child behaviour after behavioural parent training in routine clinical practice with a one group pretest–posttest design (*n* = 63). We collected outcome assessments directly before the start of the treatment (T1, pretest) and within 4 weeks after the last provided treatment session (T2, posttest), irrespective of whether the treatment was completed or not. The time between pretest and posttest was approximately 17 weeks (*M* = 16.7, *SD =* 5.7) ranging from 6 to 42 weeks.

To compare externalizing child behaviour and parental discipline practices and parenting sense of competence before treatment with a nonclinical sample, we recruited a nonclinical sample of preschoolers (*n* = 121) and their parents in the Northern region of the Netherlands.

### 2.2 Participants

The clinical sample consisted of 63 preschool children (aged 2.7‐5.9 years) with disruptive behaviour problems and their parents, participating in behavioural parent training as part of routine care. The children in this sample had at least six attention‐deficit/hyperactivity disorder (ADHD) symptoms in total or at least four symptoms in one symptom domain (attention deficit or hyperactivity/impulsivity) plus behaviour problems at home (i.e., score on the Intensity Scale of the Eyberg Child Behaviour Inventory (ECBI‐I; (Eyberg & Pincus, 1999) ≥ 132 and/or at least two oppositional defiant symptoms, as assessed with a semi–structured interview with the parents (i.e., the Dutch version of the Parent Interview for Child Symptoms PICS‐4; (Schachar, Ickowicz, & Sugarman, 2000). Participating parents gave written informed consent to use the routine care assessments for research purposes. The study had been approved by the Medical Ethical Committee of the University Medical Centre Groningen. For more details of the study procedures, see Van der Veen‐Mulders et al. (2018). Characteristics of the participating families are presented in Table 1. Although one of the secondary caretakers was a grandmother, we report on secondary caretakers as “fathers”. Compared to the sample analyzed in our previous study (Van der Veen‐Mulders et al., 2018), we could not analyze data of five families because they failed to provide T2 ratings.

**Table 1:** Baseline characteristics of children and their parents in the clinical (*n =* 63) and nonclinical (*n =* 121) sample*

	Clinical sample	Nonclinical sample
*Child characteristics*		
Male sex, *n* (%)	49 (78)	66 (55.5)
Age in years, mean (SD), range	4.6, (0.91), 2.7‐5.9	4.1, (1.00), 2.6 – 6.0
Total IQ, mean (SD), range	101, (14.39), 72‐131	
Number of ADHD symptoms	10.4, (3.69), 4‐18	
Number of ODD symptoms	2.3, (1.86), 0 – 8	
*Family characteristics*		
Highest parental education level, *n* (%)		
Low	25 (40)	18 (15)
Middle	28 (44)	34 (28)
High	10 (16)	45 (37)
Unknown		24 (20)
Single mother family, *n* (%)	16 (25)	10 (8.3)
Two parent family, *n* (%)	47 (75)	100 (82.6)
Unknown		11 (9.1)
Caucasian, *n* (%)	60 (97)	
*Maternal characteristics*		
Age in years, mean (SD), range	32.9, (4.37), 24‐41	
Biological mothers, *n* (%)	62 (98)	
Foster mother, *n* (%)	1 (2)	
*Secondary caretaker's characteristics*		
Age in years, mean (SD), range	35.8, (5.56), 24‐51	
Biological fathers, *n* (%)	41 (87)	
Stepfather, *n* (%)	5 (11)	
Foster father, *n* (%)	1 (2)	

*Note*. ADHD = attention‐deficit/hyperactivity disorder. ODD = oppositional defiant disorder. ECBI – I = Intensity Scale of the Eyberg Child behaviour Inventory. ECBI – P = Problem Scale of the Eyberg Child Inventory.

* Various information was not available on the nonclinical sample

The nonclinical sample consisted of 121 children, recruited from schools and child care centres in the Netherlands. Characteristics of the nonclinical families are presented in Table 1 as well. On the ECBI‐I, both mothers (t(180) = ‐16.4, *p* < .001) and fathers (t(122) = ‐13.3, *p* < .001) of these children stated significantly fewer disruptive behaviour problems than parents in the clinical sample. The mean mother's ECBI‐I of the nonclinical sample was 98.8 (*SD =* 22.2*,* range = 36‐158) and the mean father's score was 90.6 (*SD =* 22.2, range = 46‐146). Compared to parents in the clinical sample, mothers in the nonclinical sample also rated significantly fewer child behaviours on the ECBI Problem Scale (ECBI‐P) as troublesome, t(177) = ‐18.2, *p* < .001, as did fathers, t(114) = ‐13.6, *p* < .001. The mean mother's ECBI‐P of the nonclinical sample was 3.3 (*SD =* 5.08, range = 0‐30), and the mean father's score was 2.8 (*SD =* 4.73, range = 0‐22).

As children in the nonclinical sample were significantly younger than children in the clinical sample, *t*(182) *=* ‐3.21, *p =* .002, and consisted of significantly more girls, χ^2^(1, *n* = 184) = 9.54, *p =* .002, we matched a subsample of *n* = 63 on age and sex out of the total nonclinical sample with the clinical sample.

### 2.3 Treatment

The treatment protocol was based on two established behavioural treatments (Barkley, 1987; Forehand & Mcmahon, 1981), and the training was provided in group or individual format during twelve sessions: 2‐hour group sessions by two psychologists with a master's degree, or 1‐hour individual sessions led by one graduated psychologist. All therapists (in total 14) were experienced in delivering behavioural parent training to parents of children with behaviour problem and had received a training of 2 days specifically for the study treatment. They were supervised weekly by an experienced cognitive behavioural therapist. Supervision included discussion of therapist adherence to the treatment protocol, and therapists completed a treatment integrity checklist after each session. Furthermore, all sessions were video recorded and at random analyzed, to check adherence to the protocol. A high rate of adherence to the protocol by the therapists was found (*M* = 96% of all topics covered, SD = 70%, range 79‐100%)**.** Parents could choose the individual or group format, which led to 55 parents (87%) starting the individual format.

The primary goal of the training was to improve positive child behaviours and to reduce disruptive behaviours. In order to enable parents to modify their child's behaviours, an important secondary goal was to improve parental discipline practices and to increase parenting sense of competence. Treatment was tailored for each child, based on target behaviour problems selected by the parents in the first session, and parents learned how to observe behaviour in antecedent‐behaviour‐consequence schedules, how to improve the parent–child interaction by playing with the child, how to adjust the antecedents (e.g., setting rules, giving adequate commands, structuring the environment, anticipating new situations) and consequences (e.g. praising and using rewards, ignoring, time out, and punishment) for target behaviours, and also included maintenance training. For more details on the treatment see Van der Veen‐Mulders et al. (2018).

Parents of 70% (*n* = 44) of the participating children completed all twelve BPT sessions, while 30% (*n* = 19) stopped the treatment before the last session (range 1‐11 sessions). The mean number of sessions for the whole group was 9.9 (SD = 3.53), with a significant difference between both caregivers, t(49) = 2.26, *p* = .028, if they both participated in BPT (*n* = 47), i.e., mothers received on average 0.74 more sessions than fathers. Reasons for treatment dropout included not being able to come to the treatment on a regular basis (*n* = 9, 47%), usually because of stressful circumstances in the family. Seven families (37%) stopped because the parent training did not meet their expectations and one family (5%) because there was no need for treatment anymore. Finally, two families (11%) ended the treatment before the last session because of personal conditions.

Children whose parents stopped treatment, did not differ from the group completers in severity of externalizing behaviours before treatment, nor in total IQ. There was a greater percentage of boys (82%) in the group completers compared to the group treatment dropouts (68%).

### 2.4 Measures

Parenting outcome measures were parental total and subscale scores on the Parenting Scale (PS; Arnold, O'Leary, Wolff, & Acker, 1993) and the Parenting Sense of Competence Scale (PSOC; Johnston & Mash, 1989). The PS measures dysfunctional parenting discipline strategies and consists of a total score and two subscale scores; over‐reactivity (i.e., authoritarian, emotional and harsh parenting behaviours, and laxness (i.e., inconsistent and permissive parenting behaviours). A low PS score reveals more effective parenting behaviours. There is good evidence of internal consistency and structural validity of the PS (Blower et al., 2019).

The PSOC measures parental sense of competence on two dimensions: satisfaction, examining parents' anxiety, motivation, and frustration regarding the parenting role of the troublesome child, and efficacy, assessing parents' competence, and problem‐solving abilities in their parenting role. A high PSOC score indicates a stronger sense of competence. Acceptable levels of internal consistency and structural validity were reported on the PSOC (Blower et al., 2019).

Parental ratings on the ECBI (Eyberg & Pincus, 1999) were used to assess children's behaviour problems. The ECBI is a 36‐item inventory, rating both the intensity of problem behaviours (ECBI‐I) and if the item‐behaviour is a problem or not for the parents (ECBI‐P). Parents in the nonclinical sample completed the questionnaires at home and parents in the clinical sample at home or at the clinic. Parents were instructed to complete the measurements separately and independently. The reliability and validity of the ECBI is supported in many studies across cultures and countries (Abrahamse et al., 2015).

### 2.5 Statistical analysis

We conducted all main analyses with the total nonclinical sample (*N* **=** 121) and performed sensitivity analyses with the matched sample (*N =* 63).

To investigate differences between and within the clinical and nonclinical sample in mothers' and fathers' scores on the PS and PSOC, we used independent t‐tests. The statistical significance level was set at *p* < .05. To correct for multiple testing, we adjusted significant *p* values with the Holm–Bonferroni procedure (Gaetano, 2013). We analyzed changes in the PS and PSOC ratings in the clinical sample before (T1) and after (T2) behavioural parent training, for mothers and fathers separately, with paired *t*‐tests. Holm–Bonferroni corrections were applied per rater (mothers or fathers), for all (subscale) measures. To evaluate the clinical significance of the changes in parenting parameters between T1 and T2, we calculated effect sizes (Cohen's *d)*, by dividing the difference between two means (T1 and T2) with the pooled standard deviation. If there were guidelines available on how to deal with missing items, we followed these guidelines. In case of no such rules and less than 20% missing values for a scale, these values were replaced with the mean of other items of the scale. In total, < 0.1% scores, randomly distributed among scales, assessment time points, and participants, were missing.

In the clinical sample, changes in ECBI‐I and ECBI‐P ratings before (T1) and after (T2) behavioural parent training were analyzed with paired *t*‐tests. Following the intent‐to‐treat principle, all analyses included both treatment completers (*n =* 44) and those who had stopped the treatment before the last session (*n =* 19). To investigate whether changes in parental discipline practices and parenting sense of competence were associated with improvements of children's disruptive behaviour problems after behavioural parent training we used multiple regression analyses, with ECBI‐I and ECBI‐P T2 scores, respectively, as dependent variables and total PS and PSOC pre‐ and posttreatment difference scores as independent variables, while controlling for ECBI‐I or ECBI‐P baseline ratings. We controlled for treatment format (group vs individual) by adding treatment format as a covariate in the regression analyses. Predictors that were not significant were removed from the analyses.

Finally, we investigated the possible association between attendance rates, and changes in both parents' parental discipline practices with Pearson correlations.

## 3 RESULTS

### 3.1 Differences in parenting between parents of clinical and nonclinical children

Table 2 presents maternal and paternal ratings of parenting sense of competence and dysfunctional parental discipline strategies in the clinical and nonclinical sample. The total PS scores of parents of children in the clinical sample at baseline were significantly lower than ratings of parents in the nonclinical sample, *t*(302) = ‐6.44, *p* < .001. Also, parents' ratings on the PSOC in the clinical sample were significantly higher than parent's ratings in the nonclinical sample, *t*(279) = 8.25, *p* < .001, meaning that they experienced less sense of confidence on parenting. Regarding the subscale scores of both PS and PSOC, ratings of parents in the two samples significantly differed as well, with parents in the clinical sample reporting higher levels of lax and overreactive parenting and lower levels of satisfaction and efficacy on parenting. Focusing on ratings from mothers and fathers separately, these differences remained both on total and subscale scores, except for the PS laxness subscale; fathers in the clinical sample did not significantly differ from fathers in the nonclinical sample in ratings of permissive and inconsistent parenting.

**Table 2:** Mothers' and father's ratings on parenting sense of competence and dysfunctional parenting strategies in the clinical and nonclinical samples

Variable	Nonclinical sample				Clinical sample before treatment				t
	n	Mean	SD	Range	n	Mean	SD	range	
Mothers									
*Parenting sense of competence (PSOC)*									
Total score	107	76.9	8.26	45‐92	61	66.3	10.7	38‐89	7.16***
Efficacy	107	31.6	4.23	17‐41	61	26.8	5.41	13‐38	6.36***
Satisfaction	107	45.3	5.02	28‐54	61	39.5	6.43	21‐52	6.50***
*Parental discipline (PS)*									
Total score	117	77.0	15.1	37‐123	63	90.3	16.7	57‐127	‐5.46***
Laxness	116	22.8	6.41	11‐40	63	26.0	8.97	11‐55	‐2.49*
Over‐reactivity	116	24.1	6.85	10‐45	63	30.8	7.64	16‐53	‐5.96***
Fathers									
*Parenting sense of competence (PSOC)*									
Total score	68	77.1	9.06	52‐95	45	69.4	9.26	45‐88	4.38***
Efficacy	68	31.4	5.07	11‐42	45	27.9	5.03	17‐40	3.65***
Satisfaction	68	45.7	5.74	25‐54	45	41.5	5.98	26‐52	3.70***
*Parental discipline (PS)*									
Total score	76	77.3	16.2	43‐117	48	87.4	14.4	61‐139	‐3.53**
Laxness	76	23.9	6.47	13‐45	48	26.1	7.97	13‐48	‐1.62
Over‐reactivity	76	22.9	6.43	13‐43	48	27.3	7.20	10‐47	‐3.52**

*Note*. PS = Parenting Scale. PSOC = Parenting Sense of Competence Scale.

*t* test values, Holm–Bonferroni adjusted *p* values

* *p* < .05,

** *p* < .01,

*** *p* < .001.

Both in the clinical and the nonclinical sample, fathers and mothers did not significantly differ in mean total baseline scores on the PS and the PSOC. Regarding the subscale scores, only a significant difference between mothers and fathers was found in the clinical sample on the PS subscale over‐reactivity; mothers reported significantly higher levels of emotional and harsh discipline strategies than did fathers, *t*(109) = 2.48, *p* < .015.

### 3.2 Changes in parental discipline practices and parenting sense of competence

Table 3 presents maternal and paternal ratings of parenting sense of competence and parenting discipline strategies (PS) before (T1) and after (T2) BPT. Paired *t* tests on ratings from both parents separately revealed that significant effects of time were present for the maternal ratings on both PS, *t*(62) = 7.66, *p <* .001 and PSOC total scores *t*(60) = ‐.5.71, *p <* .001, and on all subscale scores, but fathers' ratings only changed significantly on the total PSOC score between T1 and T2, *t*(40) = ‐2.10, *p <* .042. Attendance rates were significantly related to changes in parental discipline practices, both for fathers (*r* = .45, *p* = .002) and for mothers (*r* = .259, *p* = .042): the more they were present during the sessions, the larger the changes in parenting.

**Table 3:** Mothers' and father's ratings on parenting sense of competence and dysfunctional parenting strategies before (T1) and after (T2) behavioural parent training

*Variable*	*Before treatment (T1)*				*After treatment (T2)*				*t*	Cohen's *d*
	*n*	*Mean*	*SD*	*range*	*n*	*Mean*	*SD*	*range*		
Mothers										
*Parenting sense of competence (PSOC)*										
Total score	61	66.3	10.7	38‐89	62	72.0	8.88	45‐92	‐5.71***	0.58
Efficacy	61	26.8	5.41	13‐38	62	29.5	4.61	15‐39	‐4.45***	0.54
Satisfaction	61	39.5	6.43	21‐52	62	42.6	5.38	27‐53	‐5.58***	0.52
*Parental discipline (PS)*										
Total score	63	90.3	16.7	57‐127	63	75.8	14.6	44‐114	7.66***	0.92
Laxness	63	26.0	8.97	11‐55	63	21.8	6.87	11‐44	4.76***	0.53
Over‐reactivity	63	30.8	7.64	16‐53	63	24.8	6.99	11‐43	6.72***	0.82
Fathers										
*Parenting sense of competence (PSOC)*										
Total score	45	69.4	9.26	45‐88	44	72.4	9.71	54‐94	‐2.10*	0.24
Efficacy	45	27.9	5.03	17‐40	44	29.2	5.51	19‐41	‐1.67	0.25
Satisfaction	45	41.5	5.98	26‐52	44	43.2	5.49	28‐54	‐1.53	0.30
*Parental discipline (PS)*										
Total score	48	87.4	14.4	61‐139	44	84.3	16.9	55‐121	1.19	0.20
Laxness	48	26.1	7.97	13‐48	44	25.3	8.24	11‐47	.63	0.10
Over‐reactivity	48	27.3	7.20	10‐47	44	25.7	7.25	13‐43	1.23	0.22

*Note*. PS = Parenting Scale. PSOC = Parenting Sense of Competence Scale.

*t* test values, Holm–Bonferroni adjusted *p* values

* *p <* .05,

** *p* < .01,

*** *p* < .00

Independent *t* tests indicated that maternal ratings on the total PS after BPT did not differ anymore from mothers' ratings in the nonclinical sample, *t*(178) = 0.493, *p* = .623, nor did ratings on the PS subscales. Significant differences between maternal PSOC ratings after BPT in the clinical sample and mothers' ratings in the nonclinical sample remained, both on the total score, *t*(167) = 3.57, *p* < .001, and on the PSOC subscales. After treatment, fathers in the clinical sample still differed from fathers in the nonclinical sample on all scales, except the PS subscale laxness. As mentioned before, fathers in the clinical sample did also not differ in lax parenting discipline practices from fathers in the nonclinical sample before treatment.

After behavioural parent training, mothers in the clinical sample did not differ anymore from fathers in their ratings on the PS over‐reactivity subscale. But now, mothers rated significantly more adequate discipline practices than did fathers on the total score of the PS, *t*(105) = 2.78, *p =* .007 and on the laxness subscale, *t*(105) = 2.36, *p* = .007 (see T2 scores in Table 3). Again, no differences were found between mothers and fathers after treatment regarding parenting sense of competence (see T2 scores in Table 3).

Sensitivity analyses yielded similar results, except for over‐reactivity in fathers: fathers in the clinical sample did not differ in overreactivity after parent training compared to fathers in the nonclinical sample.

### 3.3 Changes in parental discipline practices and parenting sense of competence in relation to improvements children's in children's disruptive behaviour problems after behavioural parent training

Changes of both mothers and fathers ratings on the ECBI from pre‐ to posttreatment are reported in Table 4. Mothers' ECBI‐I ratings before treatment statistically predicted ECBI‐I ratings after behavioural parent training, *R* ^2^ = 0.36, *F*(1,60) = 33.9, *p <* .001, and mothers' ECBI‐P ratings at T1 predicted their ratings at T2, *R* ^2^ = 0.18, *F*(1,60) = 13.5, *p =* .001. Improvements in overall maternal discipline practices and sense of competence on parenting added significantly to this prediction, both on ECBI‐I, *R* ^2^ = 0.51, Δ *R* ^2^ = 0.15, *F*(3,56) = 19.5, *p <* .001 and ECBI‐P, *R* ^2^ = 0.37, Δ *R* ^2^ = 0.19, *F*(3,56) = 11.2, *p <* .001. Focusing on the subscales of the PS and the PSOC, analyses revealed that only the change in maternal overreactive parenting added significantly to the prediction of the outcome variable, both on ECBI‐I, *R* ^2^ = 0.55, Δ *R* ^2^ = 0.19, *F*(2,59) = 36.10, *p <* .001 and ECBI‐P, *R* ^2^ = 0.37, Δ *R* ^2^ = 0.19, *F*(2,59) = 17.29, *p <* .001.

**Table 4:** Mothers' and father's ratings on child behaviour before (T1) and after (T2) behavioural parent training

*Variable*	*Before treatment (T1)*				*After treatment (T2)*				*t*	Cohen's *d*
	*N*	*Mean*	*SD*	*range*	*n*	*Mean*	*SD*	*Range*		
Mothers										
ECBI‐ intensity	63	157.5	24.6	89‐216	62	138.9	31.5	63‐207	‐5.57***	0.66
ECBI‐problem	63	19.2	6.36	4‐35	62	12.7	7.90	0 – 34	‐6.49***	0.91
Fathers										
ECBI‐intensity	45	146.5	24.1	94‐203	45	127.9	30.5.	62‐212	5.32***	0.68
ECBI‐problem	45	17.3	6.73	0‐31	44	11.9	8.95	0 – 36	7.71***	0.68

*Note*. ECBI = Eyberg Child Behavior Inventory

*t* test values; Holm–Bonferroni adjusted *p* values

* *p <* .05,

** *p* < .01,

*** *p* < .00

Fathers' ECBI‐I ratings before treatment statistically predicted ECBI‐I ratings after behavioural parent training, *R* ^2^ = 0.45, *F*(1,43) = 35.6, *p <* .001, and fathers' ECBI‐P ratings at T1 predicted their ratings at T2, *R* ^2^ = 0.31, *F*(1,42) = 18.8, *p <* .001. Improvements in overall paternal discipline strategies added significantly to this prediction, both on ECBI‐I, *R* ^2^ = 0.53, Δ *R* ^2^ = 0.08, *F*(2,40) = 22.8, *p <* .001 and ECBI‐P, *R* ^2^ = 0.39, Δ *R* ^2^ = 0.08, *F*(2,39) = 12.2, *p <* .001. Changes in sense of competence in fathers had no significant additional predictive value. Again, focusing on the subscales, only the change in paternal over‐reactive parenting added significantly to the prediction of the outcome variable on the ECBI‐I, *R* ^2^ = 0.57, Δ *R* ^2^ = 0.12, *F*(2,40) = 26.4, *p <* .001, but not on the ECBI‐P.

In both fathers and mothers, there were no associations between changes in the PS‐laxness, PSOC‐Efficacy, nor PSOC‐Satisfaction subscales, and changes in child behaviour.

Similar results were found for the comparisons of parental discipline strategies and sense of competence and child outcomes between the clinical sample on the one hand, and the total or the matched sample in contrast.

## 4 DISCUSSION

Our main finding was that in both fathers and mothers a reduction in authoritarian, emotional and harsh parenting after behavioural parent training was associated with improvement of the child's disruptive behaviour problems. This finding was in line with our hypothesis and with two previous studies on mediators of treatment for young children with conduct problems (Beauchaine et al., 2005; Hanisch et al., 2014). As expected, and also in line with previous studies (Ghuman et al., 2007), we found both fathers and mothers in the clinical sample reporting more inadequate parenting skills and lower parenting sense of competence before parent training than parents of children in the nonclinical sample. In a previous paper we had already reported that our behavioural parent training decreased disruptive behaviours (Van der Veen‐Mulders et al., 2018).

An overreactive parenting style can be seen as a form of parental coercion. Bor and Sanders (2004) point to parental reinforcement of negative child behaviours and vice versa (see also Lorber & Slep, 2015). Parental coercive behaviour may be one of the most crucial risk factors for future conduct problems (Bor & Sanders, 2004). Overreactive parenting models hostile behaviour, and may undermine children's self‐regulation and willingness to comply (Lorber & Slep, 2015). High levels of disruptive child behaviour problems, in turn, may elicit harsh and uncontrolled parenting disciplining practices and such inadequate over‐reactive parenting reinforces and elevates conduct problems (Lorber & Slep, 2015) . Reducing over‐reactivity in parents of young children with ADHD symptoms is especially important, as over‐reactivity has been shown to mediate the relation between early hyperactivity and later oppositional behaviours (Harvey et al., 2011). Thus, behavioural parent training appears to mitigate the risk factor of coercive parenting, especially in mothers, at least in the short run. In our study, mothers' overreactive parenting in the clinical sample clearly decreased after behavioural parent training. This finding is even more important, as maternal over‐reactivity is known to increase over time (Lorber & Slep, 2015).

Although mothers' laxness in disciplining also significantly improved after behavioural parent training, these changes were not found to be associated with changes in child behaviour, maybe due to the strong predictive value of the over‐reactivity factor. Interestingly, in contrast to parental over‐reactivity, laxness was not found to be predictive for more symptoms of oppositional defiant disorder in 3‐year old children, at the age of 6 (Harvey et al., 2011). Thus, reducing harsh and emotional maternal parenting strategies may be more important for the improvement of child behaviour problems, than reduction of permissive disciplining.

Fathers reported no improvements in the use of parental discipline practices and only a significant change in parenting sense of competence, albeit with a small effect size. Perhaps, due to the improvement of their children's behaviour, fathers experienced their parenting role as less demanding and felt themselves somewhat more competent.

A lack of effect of behavioural parent training on paternal parental discipline practices is in line with findings previous study in young children with ADHD (Webster‐Stratton, Reid, & Beauchaine, 2011), but in contrast to another study on early‐inset conduct problems (Webster‐stratton et al., 2004). Although there were only significant improvements in mothers' use of parental discipline practices, but not in that of fathers, improvement in both parents was associated with higher attendance, with a weak correlation in mothers and a moderate association in fathers. If fathers attended more sessions, paternal discipline strategies improved more.

As fathers and mothers in our sample participated in an almost equal, and high, number of treatment sessions, treatment attendance may not be a good explanation for the discrepancy between fathers and mothers regarding changes in parental discipline practices after treatment. However, all participating fathers were secondary caregivers, spending less time with their children than did mothers, and having fewer opportunities to practice new parenting skills. Perhaps, limited readiness for change may have influenced fathers' results as well. A recent study on readiness for change before starting parent training showed that fathers of young children stated less motivation to change their parenting, felt less capable to change, and regarded participating in treatment as less important than did mothers (Niec, Barnett, Gering, Triemstra, & Solomon, 2015). It may also be that fathers profit better from adapted formats of behavioural parent training, as was shown in a study on fathers of older children with ADHD (Fabiano et al., 2012). Although fathers' over‐reactivity in our study did not change significantly after treatment, there was an association between changes in paternal overreactivity and changes in child behaviour. Therefore, it may be important for therapists to choose reducing over‐reactivity as a focus of parent training when working with fathers.

Previous studies on parenting of parents of preschool children with conduct problems showed that changes in observed positive parenting strategies, but not in negative, were associated with change in children's behaviour (Gardner, Hutchings, Bywater, & Whitaker, 2010). In our study, we did not evaluate changes in positive parenting practices, but as our treatment protocol contains all these techniques and focuses on eliciting positive parent–child interactions one may hypothesize that positive parenting increased after the behavioural parent training. The interplay between positive and negative parenting may be a topic future research. For example, to investigate how treatment‐related changes in positive parenting might affect parent's use of negative parenting strategies.

Regarding parenting sense of competence, both fathers and mothers showed significant improvements after behavioural parent training, but these were not associated with improvements in child behaviour. Moreover, after the parent training parents still experienced a significantly lower parenting sense of competence than parents in the nonclinical sample. Although their child's behaviour problems had decreased, the remaining problems apparently were still demanding for both parents.

At pre‐treatment, mothers in the clinical sample reported significantly higher levels of over‐reactivity than fathers. On all the other parental parameters we found no significant differences between mothers and fathers in this group. In the nonclinical sample mothers and fathers did not differ on any of the parenting variables. As expected, parents of children in the clinical sample reported more overreactivity and lower levels of parenting sense of competence before treatment than parents of children in the nonclinical sample. Mothers in the clinical sample, but not fathers, reported more lax parenting at pre‐treatment than did mothers in the control sample.

### 4.1 Strengths and limitations

Strengths of our study were its high external validity with the embedding within clinical practice and the use of both mother and father outcome ratings on parenting. However, notable limitations were the lack of a control group, the lack of measurements of changes in parenting at various time points during the training (thus inability to evaluate mediation or directionality between parenting changes and child outcomes), the modest sample size, the fact that ratings of child behaviour and parenting were exclusively through parents' self‐report, and the lack of measures related to positive parenting. We also did not measure the extent to which mothers and fathers utilized recommended parenting strategies at home, nor if this was related to outcomes. Further research is needed to find out whether these are related.

Because of the absence of a control group, we cannot attribute the changes in maternal parenting to the behavioural parent training, although mothers discipline practices after the treatment did not differ anymore from mothers in a nonclinical sample. Some of the changes in child behaviour could be due to maturation, as the time elapsed between pre‐ and post‐treatment could be as long as 42 weeks.

Finally, the modest sample size, especially regarding fathers, may have affected study power and our negative findings regarding changes in parenting after behavioural parent training should be seen in this light.

### 4.2 Clinical implications

Findings of this study displayed that maternal parenting discipline practices and parenting sense of competence improved after behavioural parent training in an outpatient mental health clinic. In particular, the reduction of harsh and emotional parenting in both parents was related to changes in the child's behaviour. Assessment and change of uncontrolled and harsh parenting practices in both parents should thus be an important focus of any parent training program.

Although there was a high treatment attendance, fathers' parenting discipline practices did not change after behavioural parent training, maybe due to a less prominent role in daily child care and disciplining. However, assessment of readiness for change, and a pre‐treatment motivational phase, may be needed to improve outcome on paternal parenting practices. Furthermore, when fathers are involved in parent training, therapists may focus more on decreasing paternal over‐reactivity. In addition, adaptations of existing treatment protocols to the specific needs of fathers (e.g. (Fabiano, 2007) may be required. Perhaps there could be enhanced effects for both mothers and fathers by teaching specific strategies consistent with their caregiver roles.

## CONFLICT OF INTEREST

Pieter Hoekstra has received funding from Shire for a research project and has been member of a Shire advisory board meeting. Barbara van den Hoofdakker and Lianne van der Veen‐Mulders receive royalties as one of the editors of “Sociaal Onhandig” (published by Van Gorcum), a Dutch book for parents of children with PDD‐NOS and ADHD that is being used in parent training. Barbara van den Hoofdakker is and has been involved in the development and evaluation of several parent training programs, and is and has been a member of Dutch ADHD guideline groups, all without financial interests.

## FUNDING

This study was financed by The Netherlands Organization for Health Research and Development (ZonMw), grant‐number 80‐82435‐98‐9030.
